# International Prostatic Symptom Score — Voiding/Storage Subscore Ratio in Association with Total Prostatic Volume and Maximum Flow Rate Is Diagnostic of Bladder Outlet-Related Lower Urinary Tract Dysfunction in Men with Lower Urinary Tract Symptoms

**DOI:** 10.1371/journal.pone.0059176

**Published:** 2013-03-18

**Authors:** Yuan-Hong Jiang, Victor Chia-Hsiang Lin, Chun-Hou Liao, Hann-Chorng Kuo

**Affiliations:** 1 Department of Urology, Buddhist Tzu Chi General Hospital and Tzu Chi University, Hualien, Taiwan; 2 Department of Urology, E-Da Hospital, Kaohsiung, Taiwan; 3 Department of Urology, Cardinal Tien Hospital and Fu-Jen Catholic University, New Taipei, Taiwan; University of Louisville, United States of America

## Abstract

**Objectives:**

The aim of this study was to investigate the predictive values of the total International Prostate Symptom Score (IPSS-T) and voiding to storage subscore ratio (IPSS-V/S) in association with total prostate volume (TPV) and maximum urinary flow rate (Qmax) in the diagnosis of bladder outlet-related lower urinary tract dysfunction (LUTD) in men with lower urinary tract symptoms (LUTS).

**Methods:**

A total of 298 men with LUTS were enrolled. Video-urodynamic studies were used to determine the causes of LUTS. Differences in IPSS-T, IPSS-V/S ratio, TPV and Qmax between patients with bladder outlet-related LUTD and bladder-related LUTD were analyzed. The positive and negative predictive values (PPV and NPV) for bladder outlet-related LUTD were calculated using these parameters.

**Results:**

Of the 298 men, bladder outlet-related LUTD was diagnosed in 167 (56%). We found that IPSS-V/S ratio was significantly higher among those patients with bladder outlet-related LUTD than patients with bladder-related LUTD (2.28±2.25 vs. 0.90±0.88, p<0.001). TPV was similar between the two groups; however, in contrast to patients with bladder-related LUTD, patients with bladder outlet-related LUTD had higher detrusor voiding pressure, lower Qmax values, and greater postvoid residual volumes. The combination of TPV
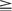
30 ml and Qmax
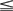
10 ml/sec had a PPV of 68.8% and a NPV of 53.5% for bladder outlet-related LUTD. When IPSS-T
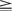
12 or IPSS-T
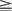
15 was considered as an additional criterion, PPV increased to 75.0% and 78.5%, respectively, and the NPV decreased to 50.9% and 50.2%, respectively. When IPSS-V/S>1 or >2 was factored into the equation instead of IPSS-T, PPV were 91.4% and 97.3%, respectively, and NPV were 54.8% and 49.8%, respectively.

**Conclusions:**

Combination of IPSS-T with TPV and Qmax increases the PPV of bladder outlet-related LUTD. Furthermore, including IPSS-V/S>1 or >2 into the equation results in a higher PPV than IPSS-T. IPSS-V/S>1 is a stronger predictor of bladder outlet-related LUTD than IPSS-T.

## Introduction

Lower urinary tract symptoms (LUTS), including voiding, storage, and post-micturition symptoms, are highly prevalent in men [1]. LUTS can result from a complex interplay of pathophysiologic features that can include bladder dysfunction and bladder outlet dysfunction such as benign prostatic obstruction (BPO), bladder neck dysfunction (BND) or poor relaxation of the urethral sphincter (PRES) [2].Urodynamically proven bladder outlet obstruction (BOO) is found in 48–53% of men with LUTS, although only 29.4% of them show evidence of BPO [2], [3].

Treatment of LUTS in men depends on the etiology of the symptoms. Traditionally, LUTS in men is usually attributed to BPO and is treated with α-adrenoceptor antagonists [4].However, men who receive treatment for prostate conditions may have persistent storage symptoms [5], [6]. Studies on LUTS in men have recently shifted from the prostate to the bladder as the source of LUTS and also as a therapeutic target [4]. Current guidelines also suggest that antimuscarinic monotherapy can be used for men with storage LUTS, those without voiding LUTS, and those without voiding BOO [7], [8], [9].

Determining the presence and the degree of BOO in men with LUTS can be difficult based on clinical symptoms alone but is important [2]. A variety of non-invasive urodynamic and non-urodynamic methods have been used to evaluate LUTS. Symptom score, urine flow rate and prostate volume are poorly predictive of BOO when used alone, and elevated postvoid residual (PVR) volume is only weakly associated with BOO [4], [10]. However, combining certain threshold values of the total International Prostate Symptom Score (IPSS-T) with maximum urinary flow rate (Qmax) and total prostate volume (TPV) may be useful for predicting BOO; however, studies have shown that this approach is not very sensitivity [10].

The IPSS consists of seven questions that deal with voiding symptoms (incomplete emptying, intermittency, weak stream and straining to void) and storage symptoms (frequency, urgency and nocturia). We previously reported that measuring IPSS subscores and calculating the IPSS voiding-to-storage subscore ratio (IPSS-V/S) is a simple and useful method for differentiating between failure to voiding lower urinary tract dysfunction (LUTD) and failure to storage LUTD [11]. The IPSS-V/S can also serve as a guide for initial treatment of male patients with LUTS.

In this study, we investigated whether IPSS-T or IPSS-V/S in association with TPV and Qmax could increase the diagnostic accuracy of bladder outlet-related LUTD in men with LUTS.

## Materials and Methods

A total of 298 men with LUTS were enrolled in the study from January 2005 to July 2010 at a tertiary teaching hospital. Men with LUTS and without documented genitourinary cancer, acute or chronic urinary retention, diabetic cystopathy, frank neuropathy, detrusor areflexia, or active urinary tract infection were included. The IPSS-voiding (IPSS-V) and IPSS-storage (IPSS-S) subscores were recorded separately by the patients using a validated Chinese version of IPSS, and the IPSS-V/S was calculated. TPV and transitional zone index (TZI) in transrectal ultrasound of the prostate, Qmax, and PVR were also evaluated.

All the enrolled patients were naïve to treatment, and the causes of LUTS were determined by videourodynamic studies (VUDS). The presence of detrusor overactivity (DO), cystometric bladder capacity (CBC), maximal detrusor pressure at Qmax (Pdet) and PVR were also recorded. VUDS were performed with a standard procedure at a filling rate of 30 ml/min with patients in a standing position and were repeated at least two times to obtain a reproducible pressure-flow tracing. The procedures and the terminology used in this study were in accordance with the recommendations of the International Continence Society unless specified otherwise [12]. Patients without an uninhibited detrusor contraction who had a strong desire to void at a capacity of <350 ml were considered to have increased bladder sensation (IBS) [13]. BPO, bladder neck dysfunction (BND), PRES, idiopathic DO (IDO), IBS, detrusor underactivity (DU) and detrusor hyperactivity with impaired contractility (DHIC) were diagnosed according to the findings of characteristic bladder dysfunction and bladder outlet dysfunction during VUDS [14].

The definition of BOO was based on the provisional International Continence Society definition of obstruction. BPO was diagnosed when a pressure-flow study showed a Pdet.Qmax >50 cmH2O or an Abrams-Griffiths number >40. In patients with equivocal pressure flow results, the features of the bladder neck, prostatic urethra, and external sphincter on voiding cystourethrography were used for the diagnosis of LUTD. Low detrusor contractility was defined as low pressure and poorly sustained detrusor contraction in combination with low urinary flow and large PVR (more than 150 ml). If sphincter electromyography showed non-relaxing activity in association with a narrow membranous urethra during voiding, the low flow rate was considered as resulting from low detrusor contractility induced by a poor relaxed urethral sphincter [2].

We divided the patients into a bladder outlet-related LUTD group, a bladder-related LUTD group, and a urodynamically normal group. Bladder outlet-related LUTD included BPO, BND and PRES, and bladder-related LUTD included IDO, IBS, and impaired detrusor contractility (including DU and DHIC). The differences in IPSS, TPV and TZI, and the differences in parameters in VUDS between the patient groups were analyzed.

Continuous variables are represented as mean ± standard deviation (SD), and categorical data are represented by number and percentage (%). Statistical comparisons between groups were tested using the chi-square test for categorical variables and the Wilcoxon rank sum test for continuous variables. Statistical assessments were considered significant when p was <0.05. Statistical analyses were performed using SPSS 15.0 statistical software (SPSS Inc, Chicago, IL). This study was a retrospective analysis and approved by Buddhist Tzu Chi general hospital research ethics committee.

## Results

The mean age of the patients was 72.7±9.0 years (range, 44–92). Bladder outlet-related LUTD was diagnosed in 167 patients (56.0%), including BPO in 88 (29.5%) patients, BND in 39 (13.1%) patients, and PRES in 32 (13.4%) patients. Bladder-related LUTD was diagnosed in 131 patients, including IDO in 77 (25.8%) patients, IBS in 41 (13.8%) patients, DU and DHIC in 7 (2.3%) patients, and in 6 (2.0%) patients in the urodynamically normal group.

Table 1 shows the differences in IPSS-T, TPV, TZI, and VUDS parameters between patients with bladder outlet-related LUTD and patients with bladder-related LUTD. We found that patients with bladder outlet -related LUTD had significantly higher IPSS-T (16.3±7.7 v 14.0±7.7, p = 0.011), higher IPSS-V (10.1±6.0 v 6.5±5.4, p<0.001), lower IPSS-S (6.2±3.6 v 7.6±3.8, p = 0.001), and a higher IPSS-V/S ratio (2.28±2.25 v 0.90±0.88, p<0.001) than patients with bladder-related LUTD. In addition, patients with bladder outlet-related LUTD had a larger TZI (43.6±16.1 v 39.1±15.0%, p = 0.017) than patients with bladder-related LUTD; however, there was no significant difference in TPV (48.9±27.3 v 43.2±23.2 ml, p = 0.058) between the two groups. Results of videourodynamic studies revealed that both groups had similar CBC values (313.5±132.9 v 285.5±146.8 ml, p = 0.089), but that patients with bladder outlet-related LUTD had higher Pdet scores (53.1±24.3 v 33.0±15.1 cmH2O, p<0.001), lower Qmax values (9.2±4.5 v 12.3±6.9 ml/sec, p<0.001), and higher PVR volumes (57.7±51.9 v 30.0±55.8 ml, p = 0.001) than patients with bladder-related LUTD.

**Table 1:** The etiologies, demographic data, IPSS, and parameters in TRUS-P and VUDS in male LUTS.

	Bladder outlet related LUTD	Bladder related LUTD	Total	P value
Patient number	167	131	298	
Age	71.8±9.3	73.8±8.4	72.7±9.0	0.065
IPSS-T	16.3±7.7	14.0±7.7	15.3±7.8	0.011
IPSS-V	10.1±6.0	6.5±5.4	8.5±6.0	<0.001
IPSS-S	6.2±3.6	7.6±3.8	6.8±3.8	0.001
IPSS- V/S	2.28±2.25	0.9±0.88	1.67±1.91	<0.001
TPV (ml)	48.9±27.3	43.2±23.2	46.4±25.7	0.058
TZI (%)	43.6±16.1	39.1±15.0	41.6±15.8	0.017
VUDS parameters				
CBC (ml)	313.5±132.9	285.8±146.8	301.3±139.6	0.089
Pdet (cmH2O)	53.1±24.3	33.0±15.1	44.3±23.0	<0.001
Qmax (ml/s)	9.2±4.5	12.3±6.9	10.6±5.9	<0.001
PVR (ml)	57.7±81.9	30.0±55.8	45.5±72.8	0.001

CBC: cystomeric bladder capacity, IPSS: International Prostate Symptom Score, IPSS-T: total IPSS score, IPSS-V: IPSS voiding subscore, IPSS-S: IPSS storage subscore, IPSS- V/S: IPSS voiding to storage subscores ratio, LUTD: lower urinary tract dysfunction, Pdet: detrusor pressure, PVR: postvoid residual volume, Qmax: maximum flow rate.

Of the 298 patients, there were 128 with TPV 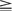
30 ml and Qmax 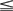
10 ml/s, among the 128 patients 88 were classified into bladder-related LUTD and the others into bladder outlet-related LUTD (Table 2). The combined criteria of TPV 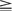
30 ml and Qmax 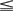
10 ml/s had a positive predictive value (PPV) of 68.8%, a negative predictive value (NPV) of 53.5%, a positive likelihood ratio (+LR) of 1.73, and a negative likelihood ratio (−LR) of 0.68 for diagnosing bladder outlet-related LUTD. When IPSS-T 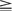
12 or IPSS-T 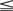
15 or IPSS-V/S>2 was included as an additional criterion, PPV and +LR increased, although the NPV slightly decreased and the −LR slightly increased. When IPSS V/S >1 was used as an additional criterion, the PPV, NPV, and +LR increased and the −LR decreased, indicating that IPSS V/S >1 in combination with TPV 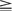
30 ml and Qmax 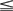
10 ml/s enhances not only the PPV but also the NPV in the diagnosis of bladder outlet-related LUTD. In addition, in comparison with IPSS-T, IPSS-V/S ratio further increased the PPV and +LR in the diagnosis of bladder outlet-related LUTD in men with LUTS.

**Table 2:** The predictive values of the combination of IPSS-T or IPSS-V/S, TPV
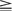
30 ml, and Qmax 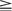
10 ml/s in the diagnosis of bladder related lower urinary tract dysfunction in men with lower urinary tract symptoms.

	Total No.	Bladder outlet-related LUTD	Bladder-related LUTD	PPV	NPV	+LR	−LR
TPV 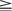 30 ml & Qmax 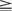 10 ml/s	128	88	40	68.8%	53.5%	1.73	0.68
+ IPSS-T 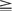 12	80	60	20	75.0%	50.9%	2.34	0.76
+ IPSS-T 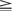 15	65	51	14	78.5%	50.2%	2.85	0.78
+ IPSS-V/S>1	70	64	6	91.4%	54.8%	8.33	0.65
+ IPSS-V/S>2	37	36	1	97.3%	49.8%	27.0	0.79

IPSS-T: total IPSS score, IPSS-V/S: the ratio of IPSS-V and IPSS-S, LUTD: lower urinary tract dysfunction, PPV: positive predictive value, NPV: negative predictive value, Qmax: maximum flow rate, TPV: total prostate volume, +LR: positive likelihood ratio, −LR: negative likelihood ratio.

## Discussion

The results of this study demonstrated that IPSS-V/S>1 is a stronger predictor of bladder outlet-related LUTD than IPSS-T. The combination of IPSS-T
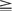
12, TPV 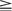
30 ml, and Qmax 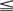
10 ml/s had a PPV of 75.0% for bladder outlet-related LUTD. When IPSS-V/S>1 was used instead of IPSS-T, the positive predictive value increased to 91.4%. Our results suggest that IPSS-V/S is a promising method for differentiating between patients with bladder outlet-related LUTD and bladder-related LUTD among men with LUTS.

BPH is one of the most common benign diseases in men with LUTS. BPH is often associated with LUTS, but LUTS generally cannot be used to make a definitive diagnosis of BPH [1]. Only about half of men with BPH will develop BPE, which may cause BOO [15]. About 25%–50% of men with histologically confirmed BPH have LUTS [16], about 60% of symptomatic men with BPH have BOO [17], and approximately 52% of asymptomatic elderly men with BPH have BOO [18]. The association among LUTS, BPH, BPE, and BOO is complex and interwoven. Clinically, the IPSS questionnaire for the evaluation of clinical symptoms, the TRUS-P for prostate size measurements, and uroflowmetry for urinary flow quantification are quick and simple methods. However, diagnosis of BOO is difficult and depends on pressure flow studies, which are expensive, invasive, and time consuming [19]. Therefore, the IPSS questionnaire, TRUS-P, and uroflowmetry are usually the first-line studies used to evaluate BOO in men with LUTS.

In our previous study, we used the area under the ROC curve to compare the diagnostic value of various non-invasive methods for predicting failure to voiding (bladder outlet-related) LUTD and failure to storage (bladder-related ) LUTD, and found that IPSS-V/S was a better predictor than IPSS-T, IPSS-V, IPSS-S, Qmax, PVR, or TPV [11]. We found that IPSS-V/S with 1 as a cut-off value had a high sensitivity and acceptable specificity for differentiating LUTD [11]. In this study, using IPSS-T as an additional criterion in the diagnosis of bladder outlet-related LUTD in men with LUTS increased the PPV by up to 14.1% (from 68.8% to 78.5%) and increased the +LR by 64.7% (from 1.73 to 2.85); however, it weakened the negative predictive values, which were primarily produced by TPV and Qmax. When the cut-off value of IPSS-V/S was set as 1, the PPV increased by up to 32.8% (from 68.8% to 91.4%) and the +LR was 4 times higher (from 1.73 to 8.33); however, this criterion also slightly increased the NPV and decreased –LR. When the cut-off value of IPSS-V/S was set as 2, the NPV was markedly lower and the –LR was higher, although it could render dramatic enhancement in PPV and +LR as was found when IPSS-V/S was set to 1. IPSS-V/S has been shown to result in better PPV and +LR than IPSS-T in the diagnosis of bladder outlet-related LUTD, and, therefore, is considered to be a better diagnostic tool in the aspect of clinical symptoms. We found that the IPSS-V/S with a cut-off value of 1 in combination with TPV
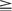
30 ml and Qmax
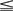
10 ml/s improved the accuracy of the positive and negative predictive values of bladder outlet-related LUTD in men LUTS.

The IPSS questionnaire has been used for decades to evaluate the severity of LUTS/BPH, and has also been applied to other conditions causing LUTS [20], [21]. However, IPSS-T correlates poorly with BOO or OAB, and is unreliable for establishing a definitive diagnosis in men with LUTS [22]. Although the clinical symptoms associated with lower urinary tract disease are not reliable for establishing a diagnosis, patients with bladder outlet-related LUTD tend to have more prominent voiding symptoms and patients with bladder-related LUTD tend to have more prominent storage symptoms. As mentioned earlier, we found in our previous study that IPSS-V/S is a more accurate non-invasive tool than IPSS-T in differentiating between these two groups of patients [11].

Pressure flow urodynamic studies remain the gold standard for establishing a diagnosis of BOO [23], [24]. Uroflowmetry and PVR are simple tests in urology clinics for evaluating the probability of BOO in men LUTS. However, either decreased urinary flow or increased PVR can result from impaired detrusor contractility or BOO. Urinary flow is unable to distinguish between these two entities without the synchronous measurement of detrusor pressure [25], and elevated PVR is only weakly related to BOO [26]. Pressure flow urodynamic studies are of value before invasive therapies in the patients with low urinary flow rate [24]. In addition, they are thought to be the only method with the potential to distinguish men with a low urinary flow rate due to detrusor underactivity from those with BOO. Although IPSS-V/S could elevate the initial diagnostic rate in differentiating bladder outlet-related LUTD from bladder-related LUTD and guide the initial treatment, it could not replace pressure flow studies in the aspect of confirmed diagnosis of BOO or the role of pre-operative evaluation.

We previously established a clinical prostate score based on the parameters of uroflowmetry and prostate measurements that is highly predictive of BPO in men with LUTS [27]. The score includes seven parameters, and we found that a prostate score of 3 points or greater had a sensitivity of 87.2% and a specificity of 60.8% for establishing a diagnosis of BPO in men with LUTS. However, the clinical prostate score is too complex to be widely used clinically. Steele GS et al investigated the value of combining symptom scores (IPSS) with urinary flow rate and prostate volume to predict BOO in men with LUTS and found that the combination had a specificity of 100% and a PPV of 100% for predicting BOO; however, the sensitivity was only 26% and the NPV was only 32% [10]. In the current study, we found that using IPSS-V/S with a cut-off of 1 in combination with TPV
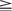
30 ml and Qmax
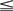
10 ml/s resulted in better positive and negative diagnostic rates than IPSS-T.

A few limitations of this study need be mentioned. First, although pressure flow urodynamic study is the standard tool for diagnosing BOO, we used VUDS to make a more accurate diagnosis in men with LUTD. Second, patients with bladder outlet-related LUTD included patients with BPO, BND, or PRES. Although LUTD was not due to BPO in all patients, this simplified classification system based on IPSS-V/S, TPV and Qmax made it easier for us to identify patients with bladder outlet-related LUTD and to give appropriate initial treatment. In addition, there could also be some inter-operator bias in the measurement of prostate volume.

### Conclusion

When used together, IPSS, TPV and Qmax increase the PPV and +LR of BOO. In comparison with IPSS-T, adding IPSS-V/S ratio >1 or >2 as a criterion further increased the PPV and +LR in the diagnosis of bladder outlet-related LUTD in men with LUTS. In addition, IPSS V/S>1 in combination with TPV 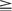
30 ml and Qmax 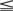
10 ml/s enhances not only PPV but also NPV in the diagnosis of bladder outlet-related LUTD. This result suggests that IPSS V/S ratio can be used to differentiate between patients with bladder outlet-related LUTD and bladder-related LUTD and that it is a more useful diagnostic tool than IPSS-T in assessing male BOO. IPSS-V/S>1 is a stronger predictor of bladder outlet-related LUTD than IPSS-T.
