# Correction: International Prostatic Symptom Score — Voiding/Storage Subscore Ratio in Association with Total Prostatic Volume and Maximum Flow Rate Is Diagnostic of Bladder Outlet-Related Lower Urinary Tract Dysfunction in Men with Lower Urinary Tract Symptoms

**DOI:** 10.1371/annotation/fd36035a-fdc9-41c1-a1f1-0fdfb146e4ec

**Published:** 2013-10-15

**Authors:** Yuan-Hong Jiang, Victor Chia-Hsiang Lin, Chun-Hou Liao, Hann-Chorng Kuo

The affiliations of the second author,Victor Chi-Hsiang Lin were not listed correctly. The correct author affiliations are as follows:

Yuan-Hong Jiang 1, Victor Chia-Hsiang Lin 2,3, Chun-Hou Liao 4, Hann-Chorng Kuo 1

1. Department of Urology, Buddhist Tzu Chi General Hospital and Tzu Chi University, Hualien, Taiwan

2. Department of Urology, E-Da Hospital, Kaohsiung, Taiwan

3. Department of Nursing, I-Shou University, Kaohsiung, Taiwan

4. Depatment of Urology, Cardinal Tien Hospital and Fu-Jen Catholic University, New Taipei, Taiwan 

